# Supplementary material for: Regulation of EGFR Endocytosis by CBL During Mitosis
Source: Cells. 2018 Dec 7;7(12):257. doi: 10.3390/cells7120257 (PMC6315415; doi:10.3390/cells7120257)
Supplement: Supplementary file 1 [file cells-07-00257-s001.pdf]

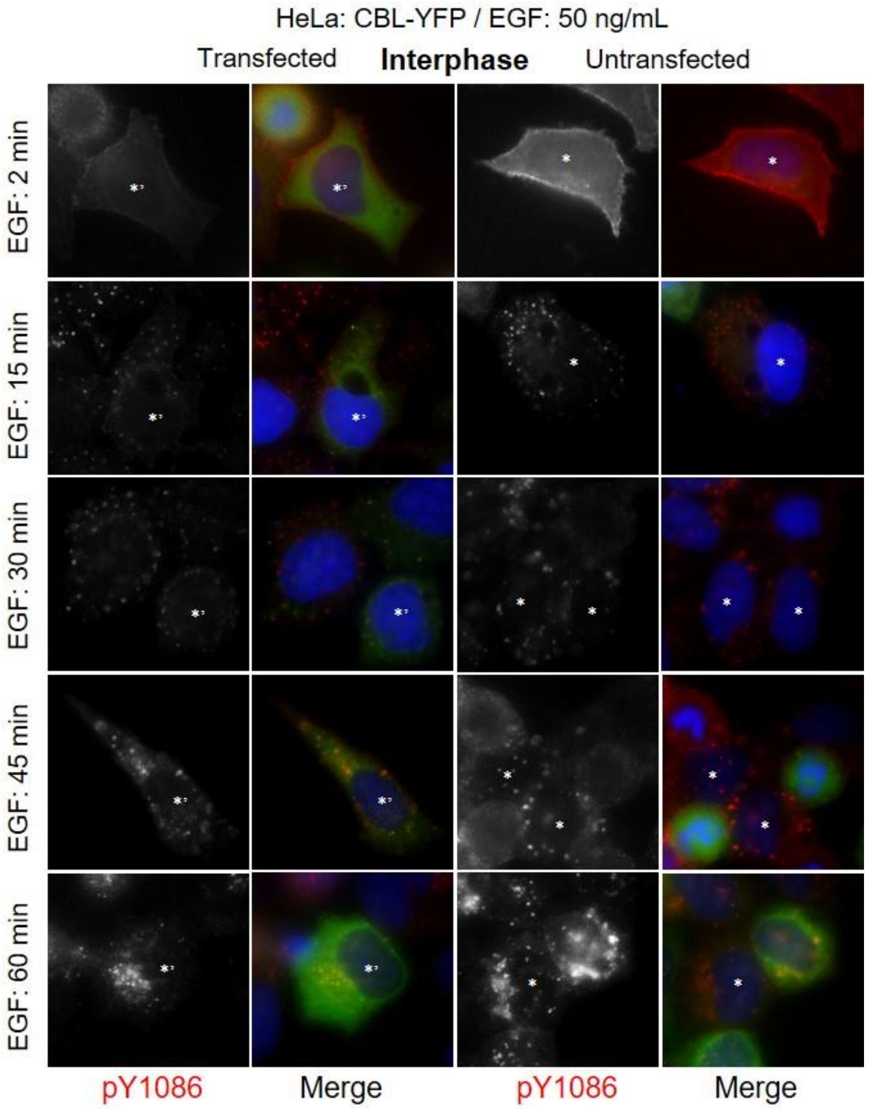

Figure S1. The effects of overexpression of CBL. Indirect immunofluorescence to observe EGFR endocytosis in HeLa cells transfected with CBL (CBL-YFP). Following the transfection of CBL, the cells were treated with nocodazole (200 ng/mL) for 16 h. The cells were then treated with EGF (50 ng/mL) for the indicated times and were stained for pY1086 (red), and DAPI (blue). The transfected cells were green. The endocytosis of EGFR in the cells transfected with wild-type c-CBL-YFP. * represents interphase cells, # represents mitotic cells, and ′ represents transfected cells.
